# Unveiling Sex-Related Variability in Psoriatic Arthritis: A Call for Personalized Care

**DOI:** 10.3390/jcm14124124

**Published:** 2025-06-11

**Authors:** Teodora Baciu, Adriana Elena Neagu, Ioana Cristina Saulescu, Daniela Opris-Belinski

**Affiliations:** 1 Department of Internal Medicine and Rheumatology, Spitalul Clinic “Sfanta Maria”, Bulevardul Ion Mihalache 37-39, 011172 Bucharest, Romania; teodora.baciu@rez.umfcd.ro (T.B.); adriana-elena.neagu@rez.umfcd.ro (A.E.N.); 2 Department of Rheumatology and Internal Medicine, Carol Davila University of Medicine and Pharmacy, 050474 Bucharest, Romania

**Keywords:** psoriatic arthritis, sex characteristics, pain measurement, comorbidity, gender, cardiovascular disease, depression, treatment outcome, precision medicine

## Abstract

**Objective:** Psoriatic arthritis (PsA) is a heterogeneous, chronic inflammatory disease that primarily affects the joints and skin, presenting with variable clinical outcomes. This review explores sex-related differences in PsA, emphasizing clinical implications, radiological manifestations, comorbidities, and tailored treatments. By examining these sex-based differences, the review aims to provide insights into how clinicians can adjust treatment strategies to better meet the distinct needs of male and female patients. **Methods:** A systematic review was conducted across national registries, interventional and observational studies, and meta-analyses including adults (≥18 years) diagnosed with PsA. The review focused on comparing clinical and radiological features, comorbidities, and treatment outcomes between sexes. A comprehensive literature search was performed in PubMed, the Cochrane Library, and Embase on 12 January 2025, with a final update on 4 April 2025. **Results:** Out of 80 records identified, 26 studies met the inclusion criteria. Women more frequently presented peripheral arthritis, reported higher pain levels, and exhibited greater functional impairment. In contrast, men showed a higher prevalence of axial involvement, radiographic progression, and more severe skin disease. Overall, treatment responses were less favorable in women, particularly with tumor necrosis factor inhibitors (TNFi), as reflected by lower rates of achieving low disease activity and reduced treatment persistence. In terms of comorbidities, data on cardiovascular risk factors were inconsistent across sexes, while depression and fibromyalgia were more frequently reported in women. **Conclusions:** Marked sex differences exist in the clinical profile, therapeutic response, and comorbidity patterns of PsA patients. Recognizing and addressing these disparities is crucial for tailoring personalized treatment strategies and optimizing patient outcomes.

## 1. Introduction

Psoriatic arthritis (PsA) is a chronic inflammatory musculoskeletal disease that falls within the broader category of spondyloarthritides and that is characterized by various patterns of joint manifestations, enthesitis, dactylitis, as well as skin and nail disease as major clinical manifestations [1]. The clinical patterns of psoriatic arthritis include distal arthritis, where the distal interphalangeal (DIP) joints are primarily involved; asymmetric oligoarthritis, affecting fewer than five small and/or large joints in an asymmetric distribution; symmetric polyarthritis, resembling rheumatoid arthritis; arthritis mutilans, a severe, deforming, and destructive form of arthritis and axial disease, involving the spine and sacroiliac joints [2]. As suggested by the name, its association with psoriasis is well documented, with up to 30% of patients with psoriasis developing arthritis, typically 5 to 12 years after the onset of their skin disease. However, in some cases, arthritis may occur simultaneously with cutaneous manifestations, and in approximately 10–15% of patients, it may precede the onset of skin lesions [3,4].

In terms of prevalence, a recent systematic review and meta-analysis reported a global rate of 112 cases per 100,000 adults, with marked geographical variation across continents [5]. Regarding gender distribution, PsA was historically considered to affect men and women equally [4,6]. However, more recent studies suggest a slight female predominance. A systematic review analyzing 481 eligible studies, including a total of 871,144 participants, reported a female majority (54.1%) in PsA prevalence. This trend was consistent across various study designs, with retrospective cohort studies showing the highest proportion of female cases [7].

Regarding the diagnosis, the CASPAR (Classification Criteria for Psoriatic Arthritis) criteria [8] require the presence of inflammatory articular disease (affecting joints, spine, or entheses) along with a minimum of three points from additional features, including personal or familial psoriasis, psoriatic nail dystrophy, negative rheumatoid factor, dactylitis, and radiographic evidence of juxta-articular new bone formation. Originally developed for use in clinical research, these criteria have since proven to be highly relevant and applicable in routine clinical practice. In a study that reviewed 480 patient records from the Royal National Hospital for Rheumatic Diseases PsA cohort, the CASPAR criteria—modified for retrospective use—demonstrated high sensitivity (99.7%) and specificity (99.1%) when compared with expert clinical diagnosis [9].

Even if not fully elucidated, the immune response in PsA involves both the innate and adaptive immune systems, with the activation of dendritic cells, macrophages, CD4+ and CD8+ T cells, neutrophils, and natural killer cells. Cytokines such as tumor necrosis factor-alpha (TNF-α), interleukin-23 (IL-23), and interleukin-17 (IL-17) play pivotal roles in driving inflammation and contributing to bone deterioration [10].

Sex-based medicine has received growing attention in recent years, prompting increased investigation into sex differences in immune-mediated diseases such as rheumatoid arthritis (RA) and axial spondyloarthritis (SpA) [11,12,13,14]. Nevertheless, in the field of PsA, current evidence remains insufficient, and further research is needed to entirely understand these disparities [14]. Sex-related differences—rooted in biological factors such as hormonal influences, immune responses, and genetic background—have been increasingly reported in psoriatic arthritis in terms of clinical presentation, treatment response, and comorbidities. Although gender—which includes sociocultural roles, behaviors, and identities—can indirectly affect health outcomes, these influences are multifaceted, variably defined, and not consistently addressed in current literature. Furthermore, gender-related sociocultural factors, such as unequal access to healthcare, stigma surrounding gender identity, and differing health-seeking behaviors, may skew results by affecting the timing of diagnoses, the initiation of treatment, and outcomes. For example, women might face delays in diagnosis due to the under-recognition of inflammatory symptoms or misattributing their symptoms to non-inflammatory issues, whereas men may hesitate to seek medical care promptly because of societal expectations around masculinity. Also, the social bias surrounding transgender patients could contribute to disparities in healthcare access and may complicate the task of isolating the impact of biological sex on clinical results. Consequently, this review primarily emphasizes sex as a biological variable, as it shows a more direct and consistently documented influence on disease mechanisms and treatment responses in psoriatic arthritis.

Integrating sex-based insights into clinical practice is essential, as recognizing and addressing these distinctions can improve disease management by facilitating earlier diagnosis, optimizing therapeutic strategies, and increasing the likelihood of achieving disease remission. This systematic review examines these differences, supporting a more personalized approach to managing disease activity and related comorbidities in PsA. The findings may contribute to the development of future clinical guidelines by advocating for sex-stratified treatment strategies and customized follow-up protocols, ultimately enhancing the quality of care for both women and men affected by the disease.

## 2. Materials and Methods

This review investigates sex-based differences in individuals diagnosed with PsA. Specifically, it explores how clinical and radiological manifestations, treatment responses, and comorbidities differ between female and male patients. The outcomes of interest included variations in clinical presentation, imaging findings, treatment response, and comorbid conditions between males and females.

A comprehensive search was conducted across electronic databases to identify relevant studies. Observational and randomized controlled trials (RCTs) and meta-analyses, as well as real-world data (RWD) from national registries reporting sex-stratified data, including adults (age ≥ 18 years) with PsA that were published between 2000 and 2025, were assessed. Studies were excluded if they did not report on sex differences in PsA, were published in a language other than English, or were conference abstracts, editorials, or case reports [15]. The searched databases are PubMed/MEDLINE, the Cochrane Library and Embase. In addition to the search process, reference lists of all included studies and relevant review articles were manually screened for additional eligible studies. The final search for all sources was completed on 4 April 2025. The results were synthesized using a narrative approach and consequently, the review protocol was not registered in PROSPERO. Due to insufficient homogeneity in outcome measures, we considered that a meta-analysis was not feasible. A comprehensive table listing the key characteristics of each included study is included in the Supplementary Materials Section (Table S1).

The search strategy was developed using relevant keywords and Medical Subject Headings (MeSH) [16] terms to identify studies examining sex differences in PsA. The following keywords were used in the search: “psoriatic arthritis”, “sex characteristics”, “male”, “female”, “gender”, “radiographic progression”, “treatment response”, and “comorbidities”. While MeSH terms were not systematically applied, Boolean operators were used to refine the results. Search strings reflecting the practical approach taken across PubMed, Embase, and the Cochrane Library are provided in the Supplementary Materials.

**Figure 1:**
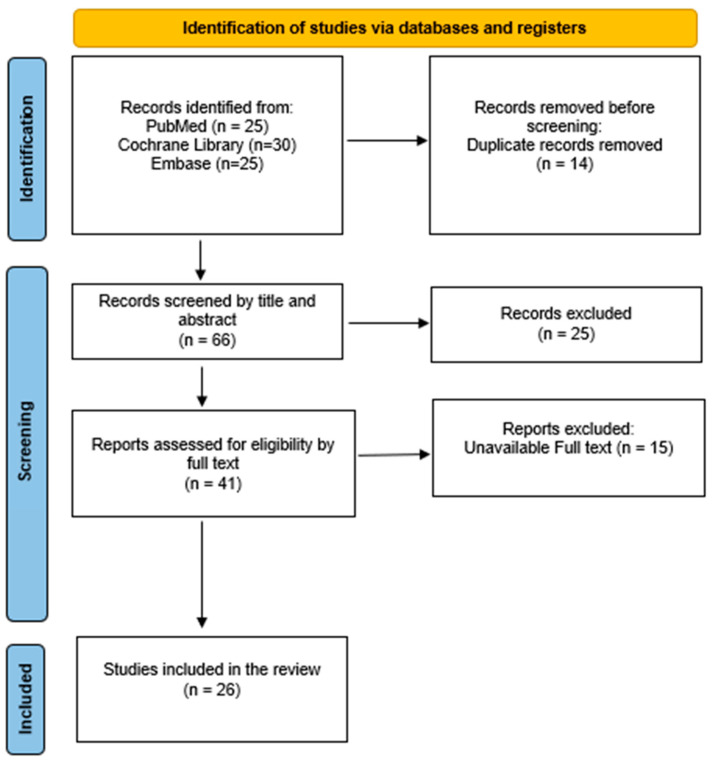
Preferred Reporting Items for Systematic Reviews and Meta-Analyses (PRISMA) flowchart.

Three independent reviewers conducted the study selection process. Data were extracted using a standardized format to capture study characteristics and outcomes relevant to sex-based differences in psoriatic arthritis. Titles and abstracts of all identified records were screened for eligibility. Full-text articles were subsequently assessed for inclusion. Any discrepancies between reviewers were resolved through discussion, and, if necessary, consultation with a fourth reviewer. The study selection process is illustrated using a PRISMA 2020 flow diagram [17] (Figure 1).

Extracted information included study characteristics (author, year, country, study design), population demographics, clinical and radiological features stratified by sex, treatment outcomes, and comorbidities. Data were organized thematically based on the primary outcomes of interest.

Due to the use of diverse outcome measures for disease activity across included studies, we accounted for variability in disease activity measures by discussing findings based on the tools used—such as the Disease Activity Score using 28 joint counts (DAS28) [18], the Clinical Disease Activity Index for Psoriatic Arthritis (cDAPSA) [19], and the Minimal Disease Activity (MDA) criteria. As highlighted by Linde et al. [20], differences between activity scores—such as higher remission rates with DAS28 compared to cDAPSA—can influence interpretation. By presenting results within the context of each measure, we aimed to qualitatively assess consistency across studies. This approach helped mitigate the impact of methodological heterogeneity. Where reported, effect sizes and corresponding confidence intervals were extracted to enable more precise comparisons between sexes. The methodological quality of all studies was evaluated using an abbreviated version of the *Standard Quality Assessment Criteria for Evaluating Primary Research Papers from a Variety of Fields* developed by Kmet et al. [21]. The quality assessment was conducted by one reviewer, with all judgments independently verified by a second reviewer to ensure accuracy and minimize potential bias. A summary of the quality assessment findings is provided in Supplementary Table S2. Findings were grouped and reported according to three main themes: clinical manifestations, the impact of comorbidities, and treatment responses in male and female patients with PsA.

This systematic review was designed and reported following the Preferred Reporting Items for Systematic Reviews and Meta-Analyses (PRISMA) 2020 statement. A PRISMA flow diagram was used to depict the study selection process, and a completed PRISMA checklist is provided [17].

## 3. Results and Discussion

The main results concerning the sex-differences in the clinical and radiological presentation, treatment response, and comorbidities are summarized in Table 1.

### 3.1. Clinical and Radiological Differences

From a clinical perspective, notable sex-related differences have been observed in disease presentation. A systematic review by Coates et al. [22], which included 27 studies, emphasizes a higher prevalence of peripheral involvement in women, particularly in terms of the number of painful joints and functional limitations. However, data on the number of swollen joints were mixed, with some reports indicating an equal number of swollen joints between sexes. This fact can be attributed to differences in pain perception in women [31,32], leading to a higher reported rate of peripheral disease. Conversely, axial involvement in psoriatic arthritis was more frequently observed in males. In the context of peripheral psoriatic arthritis, although this type of articular involvement is more prevalent in females, it also occurs in males. Among the patterns of peripheral involvement, the polyarticular subtype is more common in females [31,33], whereas oligoarticular involvement and DIP joint disease are more frequently observed in males [23]. The latter is particularly associated with developing progressive erosive bony lesions [34]. A potential explanation for the greater involvement of DIP joints in males may lie in its association with nail disease, which is also more frequently observed in the male population [22,35]. This association has been previously demonstrated in magnetic resonance imaging (MRI) studies [36]. The involvement of DIP joints in psoriatic arthritis secondary to nail disease is primarily attributed to the anatomical and pathological relationship between the nail unit and the enthesis, as the nail apparatus is closely associated with the extensor tendon enthesis at the DIP joint [37].

Beyond articular involvement, enthesitis appears to be more prevalent among women with PsA. This finding has been linked to poorer patient and physician-reported outcomes, underscoring the importance of routinely assessing this clinical domain during medical visits. Evidence that supports this observation comes from clinical studies employing tools such as the Maastricht Ankylosing Spondylitis Enthesitis Score (MASES) [38] as well as from data reported in national registries [22,39]. Concerning dactylitis, the sex-specific prevalence remains unclear based on current evidence. However, this condition appears to be associated with higher disease activity and poorer functional status, similar to enthesitis [22,40].

Concerning the rate of skin involvement (psoriasis), Coates et al. observed a tendency for a more severe burden in men compared to women, as measured by the Psoriasis Area and Severity Index (PASI) [41]. Similarly, a multicenter Turkish study involving 1038 patients (678 females, 360 males) of similar age, classified as peripheral PsA according to the CASPAR criteria [8], compared disease activity scores and patient-reported outcomes between sexes. Higher PASI scores and greater axial involvement were observed in men [42].

As for other extra-musculoskeletal manifestations, such as uveitis and inflammatory bowel disease, comprehensive data explicitly addressing sex differences in prevalence or clinical expression remain limited [22,42].

Focusing on the radiological aspect, Eder et al. [23] conducted a cross-sectional analysis of 345 men and 245 women, finding that axial involvement was more frequent in men than women with PsA (42.9% vs. 31%, *p* = 0.003), even after adjusting for duration of PsA (OR = 1.8, 95%CI 1.3 to 2.6, *p* = 0.002). Radiographic damage was assessed using the modified Steinbrocker score (mSS) for 42 peripheral joints [43], the modified Stokes Ankylosing Spondylitis Spine Score (mSASSS) [44] for radiographic damage in the lumbar and cervical spine, and the New York criteria [45] for defining sacroiliitis. Interestingly, in addition to exhibiting a higher rate of radiographic axial damage characterized by grade 3 or 4 sacroiliitis and syndesmophytes formation in the cervical, thoracic, and lumbar spine, male patients also showed an increased likelihood of developing radiographic damage in peripheral joints. In contrast, women were more likely to have non-erosive disease (mSS = 0). Despite this, they experienced greater functional impairment and a poorer quality of life. These findings may be influenced by a range of factors, including sex hormones, environmental exposures (such as trauma and physically demanding occupational activities) and differences in therapeutic approaches. Notably, women had a higher consumption of conventional synthetic disease-modifying antirheumatic drugs (csDMARDs), which may contribute to less radiographic progression over time, although the protective role of csDMARDs in preventing structural damage remains a matter of ongoing debate.

Several pathophysiological mechanisms have been proposed to explain the differences in peripheral and axial involvement between men and women. A primary factor is the difference in genetic predisposition. Men with PsA are more likely to carry the human leukocyte antigen-B27 (HLA-B27) allele, which is strongly associated with axial disease involvement. Queiro et al. [46] showed that the presence of this genetic marker is linked to an earlier onset of both psoriasis, bilateral sacroiliitis, and a higher prevalence of uveitis, conditions that seem to be more frequently observed in men. The pathophysiological mechanisms by which HLA-B27 contributes to axial disease involve its ability to present arthritogenic peptides to CD8+ T cells, thereby initiating an autoimmune response through a mechanism of molecular mimicry. Additionally, HLA-B27 may misfold within the endoplasmic reticulum, triggering an unfolded protein response that leads to increased inflammation through the production of pro-inflammatory cytokines, such as interleukin-23 (IL-23) and interleukin-17 (IL-17). Another mechanism could be that HLA-B27 may exhibit aberrant properties, including its ability to form cell-surface dimers that interact with innate killer cell immunoglobulin-like receptors (KIRs), such as KIR3DL2, on CD4+ Th17 cells. This interaction promotes the production of pathogenic cytokines, thereby driving inflammation [47].

Secondly, hormonal differences may play a significant role. Estrogen and testosterone exert distinct effects on the prevalence and severity of psoriatic arthritis, particularly concerning peripheral versus axial disease involvement. Estrogen has immunomodulatory effects, which may contribute to an inflammatory response. During pregnancy, estrogens appear to have a dual role in immune regulation. They promote T helper 1 (Th1)-driven cell-mediated immunity at low concentrations by enhancing interferon-gamma (IFN-γ) production in the uterus and in vitro. In contrast, higher concentrations of estrogens induce the expansion of regulatory T lymphocytes (Treg), which are essential for maintaining immune tolerance and preventing autoimmunity. Conversely, progesterone contributes to immune homeostasis by modulating both Th1/Th2 and Th17/Treg responses. It promotes Treg cell expansion while inhibiting Th17 cell differentiation—an effect crucial for establishing maternal–fetal tolerance during pregnancy [48,49]. Moreover, fluctuating estrogen levels in premenopausal women appear to be associated with increased production of pro-inflammatory cytokines such as TNF-α and interleukin 6 (IL-6), which may exacerbate peripheral joint inflammation. On the other hand, androgens modulate Th17 cell metabolism—specifically glutaminolysis—thereby reducing Th17-mediated inflammation. Additionally, they promote the differentiation and function of Treg lymphocytes, enhancing their suppressive capacity and contributing to immune regulation [49]. In contrast, lower testosterone levels have been linked to increased disease severity [50]. Furthermore, women seem to have stronger innate and adaptive immune responses than males [51].

### 3.2. The Impact of Comorbidities

Comorbidities are recognized as a key factor contributing to the overall burden of PsA, influencing both disease progression and treatment outcomes. A systematic review and meta-analysis conducted by Gupta et al. [52], which included 39 studies with a combined sample size of 150,677 patients diagnosed with PsA, reported that the most prevalent comorbidities were hypertension (pooled prevalence 34%), metabolic syndrome (29%), obesity (27%), hyperlipidemia (24%), and cardiovascular diseases (19%). Cardiovascular mortality is significantly higher among patients with rheumatic diseases (rheumatoid arthritis, psoriatic arthritis, and ankylosing spondylitis) compared to the general population [53,54]. These patients have a markedly increased risk of cardiovascular events, including acute coronary syndromes, coronary artery disease, stroke, and thrombotic events [55]. Furthermore, data from a cross-sectional study analyzing 518 patients with PSA and non-PsA SpA showed an increased incidence of diabetes (OR = 1.35, 95% CI 1.17–1.56), hyperlipidemia (OR = 1.60, 95% CI 1.46–1.75), hypertension (OR = 1.81, 95% CI 1.66–1.97), coronary artery disease (OR = 1.39, 95% CI 1.19–1.64), hyperuricemia (OR = 1.61, 95% CI 1.39–1.86), and metabolic syndrome (OR = 1.58, 95% CI 1.32–1.88), with *p*-values all less than 0.001 in patients with PsA. Moreover, different components of the metabolic syndrome (i.e., waist circumference and triglyceride level) were also significantly different between the two groups [56]. One possible etiopathogenic explanation may be linked to the immune response which involves enhanced activation of T cells and myeloid cells, platelet activation, and up-regulation of inflammatory cytokines such as interferons, TNF-α, and interleukins (IL-23, IL-17, and IL-6), these being linked to vascular inflammation and atherosclerosis development [57,58].

A literature review by Atzeni et al. [24] reported conflicting findings on sex differences in cardiovascular risk factors and mortality in patients with psoriatic arthritis, with significant variations in the prevalence of hypertension, dyslipidemia, and diabetes mellitus between sexes across different populations. However, an analysis of the German BARMER health insurance database [25], comprising 11,984 individuals with PsA, receiving DMARD therapy in 2021 and 119,840 age- and sex-matched controls, revealed that men with PsA more frequently exhibited dyslipidemia (36% vs. 31%), cardiovascular disease (21% vs. 14%), and gout (8% vs. 2.7%) compared to their female counterparts [25]. Another epidemiological survey conducted by the Japanese Society for Psoriasis Research [26] from 2017 to 2020 reported that the most common comorbidities were hypertension (men 39.6%, women 29.2%), dyslipidemia (men 22.9%, women 16.8%), diabetes mellitus (men 21.5%, women 14.9%), hyperuricemia (men 19.8%, women 2.2%), cardiovascular disease (men 5.0%, women 2.5%), and cerebrovascular disease (men 4.1%, women 3.4%), also showing a tendency toward higher prevalence in men.

The data on the prevalence of cardiovascular events also demonstrated a predominance in males, with the notable mention that the male sex is considered a non-modifiable risk factor. A British cohort study of 709 patients with severe PsA initiating TNFi therapy found increased all-cause mortality, mainly due to excess cardiovascular deaths [29]. Standardized mortality rates were elevated for cardiovascular disease (1.89; 95% CI 1.01–3.24) and coronary heart disease (2.42; 95% CI 1.11–4.59), reaching significance only in men (2.80; 95% CI 1.13–5.78) [59]. Fragoulis et al. demonstrated that, among 215 patients with PsA recruited cross-sectionally from two tertiary care hospitals, major adverse cardiovascular events (MACEs) were associated with male sex [odds ratio (OR) for females: 0.26; 95% confidence interval (CI): 0.06–1.23] and hypertension [OR (95% CI): 6.07 (1.12–33.0)].

Variations in age distribution, disease duration, and lifestyle factors—such as smoking, diet, and physical activity—can influence cardiovascular risk and often differ by sex but are not consistently reported. Socioeconomic disparities and unequal access to healthcare may affect the detection and management of comorbidities, introducing bias. Additionally, hormonal differences, including the loss of estrogen’s protective effects after menopause, heterogeneity in study design, and regional cardiovascular epidemiology, further complicate comparisons. Together, these factors underscore the need for standardized, sex-stratified research.

Regardless of sex, clinicians should assess cardiovascular risk following the 2016 EULAR recommendations [58], stratifying risk at least every five years. Based on this assessment, appropriate measures should include lifestyle modifications, antihypertensive therapy, lipid-lowering medication, and, whenever possible, tapering corticosteroids and limiting nonsteroidal anti-inflammatory drugs (NSAIDs). Moreover, a cardiovascular assessment of the patient is essential for managing comorbidities and guiding treatment decisions. For instance, the use of TNFi is contraindicated in patients with congestive heart failure [60], while Janus kinase inhibitors (JAKi) are not recommended in patients with a history of cardiovascular events, particularly myocardial infarction or stroke, and should be used with caution in individuals over 65 years old with established cardiovascular risk factors [60,61,62].

For decades, obesity has been recognized not only as a significant modifiable cardiovascular risk factor but also as a comorbidity that negatively impacts disease activity. Interestingly, in PsA, obesity was associated with higher baseline disease activity and diminished response to TNFi according to data pulled from an observational cohort study of 1943 PsA patients, based on the Danish and Icelandic biologics registries. Obese patients had higher baseline DAS28, levels of inflammation, and visual analog scale (VAS) pain scores compared to non-obese patients. Additionally, the median duration of TNFi therapy was significantly shorter in obese patients (2.5 years; 95% CI 1.7–3.2) compared to non-obese patients (5.9 years; 95% CI 4.1–7.7; *p* < 0.01) [63]. Concerning its sex prevalence, the available data are mixed. While increasing age and female sex appear to be risk factors for obesity in patients with psoriasis and PsA [64], a retrospective study which included 132 patients with a confirmed diagnosis of PsA according to the CASPAR criteria [8] reported only a tendency (*p* = 0.06) for higher obesity rates in women with PsA compared to men [65]. Coates et al. [22] noted that among ten studies reporting BMI values, only two demonstrated significantly higher BMI in females than in males. An interesting aspect regarding sex differences and obesity in patients with psoriatic arthritis is that literature data suggest weight loss may lead to a reduction in IL-23 levels, which positively correlates with lower disease activity scores and C-reactive protein (CRP) levels in women but not in men. Furthermore, this suggests that weight management strategies could play a more critical role in improving disease outcomes for female patients with psoriatic arthritis, highlighting the need for a tailored approach to managing obesity-related inflammation [66].

In terms of psychological impact, women appear to experience greater pain, functional disability, fatigue, lower overall well-being, and a higher disease burden. Females also seem to have higher rates of depression and fibromyalgia [22,42]. Albrecht et al. additionally supported in the analysis of the German BARMER health insurance database [25] that depression is significantly more prevalent in females, with 32% of women affected compared to 21% of men. Also, in a cross-sectional study by Haque et al. [56], comparing comorbidities in PsA and non-PsA SpA, depression was found to be significantly more prevalent in women than in men (*p* < 0.001), regardless of the SpA subtype. Similarly, a 2014 study published in the Journal of Rheumatology that compared 306 patients with PsA and 135 with PsC (psoriasis without PsA), found that prevalence of both anxiety and depression was higher in patients with PsA (36.6% and 22.2%, respectively) compared to those with PsC (24.4% and 9.6%; *p* = 0.012, 0.002), with higher rates being observed in females. The study also noted that depression in PsA patients was associated with factors such as unemployment, higher actively inflamed joint count, disability, pain, and fatigue [67]. Duruöz et al. [42] also demonstrated on a group of 1038 patients with PsA that female patients had significantly higher anxiety, depression, fibromyalgia, and fatigue scores than males (*p* < 0.05).

Drawing parallels with other inflammatory conditions, a study of 83 PsA patients and 199 RA patients found that 25.1% of RA patients reported moderate to severe depressive symptoms, compared to 21.7% of PsA patients, indicating a slightly higher prevalence in RA [68,69]. However, in a cohort of 114 patients with PsA and 201 patients with RA who attended the Department of Rheumatology and Clinical Immunology at Peking University First Hospital over 15 months [70], PsA patients had 2.7 times higher odds of experiencing depression compared to RA patients, highlighting a contrasting trend. Additionally, a 2020 multicenter study reported higher rates of depression among PsA patients compared to those with RA and diabetes mellitus [71]. Interestingly, depression was associated with female sex [OR (95% CI): 3.47 (1.51–7.99)].

Although the data show that depression is more prevalent in women with PsA, it is important to acknowledge that depression in men may often be underdiagnosed due to societal stigma and differing symptom presentations [72]. Men are more likely to exhibit externalizing behaviors such as irritability, anger, or substance use, which may not be immediately recognized as signs of depression by healthcare providers. Additionally, societal norms often discourage men from seeking help or expressing emotional distress, which may further contribute to underdiagnosis.

It is important to mention that the variability in comorbidity data across studies likely reflects differences in study populations, geographic regions, and diagnostic criteria. Methods of data collection—such as self-reported versus clinically validated diagnoses—also vary, as do definitions of conditions like obesity and depression. Sex- and age-related physiological differences, healthcare access, and health-seeking behaviors further complicate interpretation. Standardized definitions and harmonized data collection are needed to improve comparability and clarify sex-based differences.

### 3.3. Differences in Treatment Response

The main data regarding various therapeutic agents in PsA and the associated sex-related differences in outcomes are summarized in Table 2.

In addition to differences in clinical manifestations, pathogenesis, and comorbidities, gender-based variations in treatment response rates have also been observed. According to guidelines [61] the actual treatment of psoriatic arthritis includes NSAIDs, csDMARDs such as methotrexate, leflunomide, and sulfasalazine, as well as biologic DMARDs (bDMARDs), including TNFi, IL-17 inhibitors (IL-17i), IL-12/23 inhibitors (IL-12/23i), and IL-23p19 inhibitors (IL-23p19i). Additionally, targeted synthetic DMARDs (tsDMARDs) such as JAKi or phosphodiesterase 4 inhibitors (PDE4i) are available. Treatment choice depends on the predominant axial or peripheral involvement pattern and non-musculoskeletal manifestations [50]. However, until now, no gender-specific recommendation has been made.

TNFi are widely used biologic agents due to robust safety data, extensive rheumatologist experience, and a lower economic burden attributable to the availability of biosimilars [73]. A detailed overview of sex-related differences in treatment outcomes with TNF inhibitors, as well as other biologic therapies, is presented in Table 2. In this context, the European Spondyloarthritis Research Collaboration Network conducted a study using data from 7679 patients with PsA—with an equal distribution of women and men—who initiated their first TNFi across 13 European registries. The primary outcome was achieving low disease activity (LDA) at six months, as measured by DAS28-CRP. The study found that a lower percentage of women reached LDA compared to men. Furthermore, when evaluating TNFi retention in a larger cohort of 17,842 patients, women exhibited significantly lower retention rates than men at 6, 12, and 24 months [29]. Similarly, a prospective, real-world observational cohort study involving 929 patients with psoriatic arthritis (512 females and 417 males) who initiated ustekinumab (an IL-12/23 inhibitor) or a TNFi as a first-, second-, or third-line bDMARD demonstrated that women generally experienced poorer responses than men across both treatment groups. Despite having more severe disease at baseline, women presented worse patient-reported outcomes at 12 months and exhibited lower treatment persistence compared to men [33].

Furthermore, exploring the data on IL17i, a subgroup analysis using pooled data from patients enrolled in the SPIRIT-P1 [74] and SPIRIT-P2 [75] trials showed that male patients had greater treatment response rates in terms of activity scores, although the clinical significance is unclear [27]. At baseline, female patients were older, had a higher BMI, and exhibited worse scores in tender joint count (TJC), HAQ-DI, and Leeds Enthesitis Index (LEI) [76], despite having lower CRP levels compared to male patients. After 156 weeks of treatment with ixekizumab, women reported less improvement in patient-reported outcomes (PROs), particularly in pain, Patient Global Assessment (PtGA), and HAQ-DI. Several factors have been suggested to contribute to these sex-related differences. Variations in weight and BMI could influence physical function (as measured by HAQ-DI), the pain visual analog scale (VAS), and PtGA scores, and may also affect drug pharmacokinetics, potentially impacting treatment response. Additionally, menopause could influence joint-related symptoms and pain perception, thereby affecting pain VAS and PtGA scores. Functional outcomes may also be impacted by age, obesity, comorbidities, and hormonal status. Furthermore, implicit bias in clinical practice—such as a tendency to downplay the symptoms reported by female patients—may contribute to undertreatment or discrepancies in physician-assessed outcomes [27,77].

Regarding JAKi, a post hoc analysis of two phase 3 randomized controlled trials of Tofacitinib in psoriatic arthritis reported comparable ACR20/50/70 response rates between sexes. However, female patients were less likely to achieve MDA because of the different baseline levels of disease expression. The safety profile and treatment retention rates were similar [30].

Concerning the influence of gender on treatment persistence, a recent cohort study [28] that included 14,778 patients included in the healthcare database of the French health insurance scheme who were new users of the targeted therapy found a significant sex difference in treatment retention between sexes (lower for women than men) for TNFi and IL17i but not for IL12/23i, IL23i, or JAKi. Moreover, women had higher healthcare consumption than men, with more use of csDMARDs, NSAIDs, and prednisone but also more analgesics, corticosteroid injections, hospitalizations related to PsA, and consultations with a rheumatologist.

Several potential explanations have been proposed for the observed differences in treatment response between female and male patients. Besides the effects of sex hormones which were already discussed [50,78], the genetic component may also play a key role, taking into account that the X chromosome contains numerous immune-related genes, and its incomplete inactivation in females can lead to overexpression of these genes, potentially enhancing immune responsiveness and increasing susceptibility to autoimmune conditions like PsA [50]. Race and socio-economic status could be major contributors to the evaluation of treatment response. An analysis of U.S. patient data indicated that Caucasian patients were more likely to be treated with TNFi (51% vs. 41%), while African American patients had higher usage of csDMARDs (98% vs. 72%). These differences may reflect variations in treatment access, preferences, or provider prescribing behaviors [79]. On the same note, data from the National Health and Wellness Survey highlighted that racial and ethnic minority patients with PsA reported poorer health-related quality of life and greater work productivity loss compared to white patients, underscoring the broader impact of PsA on minority populations [80].

Additionally, women may have increased sensitivity to pain, which could contribute to higher levels of reported disability [31]. A meta-analysis of individual patient data from 33,957 participants enrolled in 10 large international randomized controlled trials involving patients with diverse disease states demonstrated that women reported pain more frequently and with greater intensity than men, regardless of age or geographic region [32]. Another systematic review, designed to summarize findings from a decade of human laboratory research on sex and gender differences in pain perception using a comprehensive electronic search strategy, demonstrated that women have lower pain thresholds and exhibit reduced tolerance to thermal and pressure pain compared to men, although thresholds for cold and ischemic pain were comparable [81]. The differences in pain perception between sexes seem to have a multifactorial background, involving biological, psychological, and social factors. Pain intensity perception appears to correlate positively with anxiety levels among women, who are known to exhibit higher overall anxiety sensitivity than men [82]. Additionally, women seem to experience central sensitization phenomena more frequently than men—a mechanism implicated in the chronicity of pain, even in healthy individuals [83]. The hormonal profile could play a significant role in pain perception, although the underlying mechanisms remain unclear [22,84]. However, some mice studies have highlighted the protective role of testosterone in chronic pain [22,85]. Moreover, the higher reported rate of fibromyalgia among women with PsA may represent a potential bias in the perception of increased pain intensity within the context of the disease [22,33].

Compared to women, men generally have greater muscle strength, which may facilitate daily functioning and influence scores on functional assessments such as the HAQ-DI. Furthermore, higher muscle mass and strength in men may confer greater resilience following improvements in joint inflammation [23,27]. Moreover, existing literature indicates that in rheumatoid arthritis, women tend to report higher levels of pain, greater disease activity, and worse disability scores compared to men, who typically have greater muscle strength [86]. On the other hand, disparities in psychosocial factors influencing treatment response may be related to gender-based differences in healthcare-seeking behavior shaped by societal norms. This may contribute to a differential perception and underreporting of adverse events among men compared to women [28,87]. Furthermore, a systematic review has reported that women may exhibit lower adherence to therapy compared to men, although this difference tends to diminish with treatments that have longer dosing intervals or are administered orally [28,88]. It is also possible that healthcare professionals may perceive women as being more sensitive to pain or as exaggerating their symptoms, which can lead to underdiagnosis or an underestimation of disease activity. This is a critical issue, and increasing awareness within the healthcare system regarding how pain is reported in PsA is essential for improving patient assessment and management. On the other hand, a greater level of reported pain in women could also help explain the lower treatment retention rates observed in female patients, as inadequate symptom control may lead to treatment discontinuation [89]. Additionally, sex-based differences in expectations, particularly regarding daily functioning and skin involvement, may also play a role, potentially contributing to the observed variation in treatment persistence across therapeutic classes [88]. Overall, the mechanisms underlying differential treatment responses between sexes are multifactorial and remain incompletely understood. Pharmacokinetic differences—such as drug absorption, distribution, metabolism, and clearance—may vary between men and women, potentially affecting efficacy and side-effect profiles. Hormonal influences can modulate immune responses, particularly during key life stages like menopause or pregnancy. Although direct evidence linking hormonal biology to treatment response is scarce, several mechanistic hypotheses can be proposed. As previously noted, fluctuating estrogen levels in premenopause appear to be associated with increased production of pro-inflammatory cytokines (TNF-α, IL-6). Subsequently, in postmenopausal women, the loss of estrogens leads to further elevation of IL-1, IL-6, IL-17A (by a higher ratio of Th17/Treg), and TNF-α, potentially contributing to reduced treatment response [48,90,91]. In this context, treatment considerations for postmenopausal women with PsA should include the potential need for medication adjustments and the consideration of new therapies to effectively manage changes in disease activity [92]. In contrast, during pregnancy, higher concentrations of estrogens induce the expansion of Treg, potentially explaining the slight improvement in disease activity observed during this period [91]. Conversely, the downregulation of Th17-mediated inflammation and the promotion Treg function by androgens [49] may partly account for more favorable treatment outcomes in males. Psychosocial factors also play a role: disparities in healthcare utilization, health literacy, and trust in healthcare providers may influence adherence and persistence. Addressing these gaps requires an integrative approach that combines biological, pharmacological, and socio-behavioral perspectives in study design and clinical practice. In this context, there is a strong rationale for developing sex-specific treatment algorithms in PsA by incorporating factors such as hormonal status, pain sensitivity, and pharmacokinetics, to enhance treatment effectiveness and adherence.

This research has some limitations because many studies include relatively small sample sizes, which reduces statistical power and limits the ability to detect meaningful sex-specific differences. More importantly, the majority of clinical trials and observational studies in psoriatic arthritis do not incorporate sex as a core variable in their design. There is a striking lack of randomized controlled trials that are specifically stratified by sex or that perform predefined, sex-specific subgroup analyses. This omission not only restricts our understanding of how disease pathophysiology, progression, and treatment responses may differ between men and women but also risks overlooking key clinical differences that could inform more tailored therapeutic strategies. Additionally, this review included only studies published in English, which may have introduced language bias and limited the scope of international data considered. This exclusion may also affect the generalizability of our findings, particularly with respect to sex differences that could be influenced by cultural, societal, or healthcare system-related factors. Furthermore, selection bias in registries could represent a concern, as they often exclude severe cases requiring hospitalization, leading to the underrepresentation of patients with advanced disease. This may result not only in an underestimation of the comorbidity burden but also in an overestimation of treatment effectiveness, as patients with milder disease are generally more responsive to therapy. These limitations may also skew observed sex-based differences and reduce the applicability of the results to real-world clinical settings.

To address these gaps, future research must prioritize better-designed studies that ensure balanced sex representation, systematically stratify outcomes by sex, and include analyses that explore sex as a biological and clinical modifier. These efforts are essential to develop truly personalized approaches in psoriatic arthritis and to promote equity in both clinical research and patient care.

## 4. Conclusions

Sex-based medicine plays a crucial role in the management of psoriatic arthritis, as significant differences in pathogenesis, clinical presentation, and response to treatment have been identified between men and women. Understanding the clinical differences between sexes in psoriatic arthritis is crucial for achieving earlier and more accurate diagnoses, improving treatment outcomes, and promoting personalized care. By recognizing the distinct clinical pattern between men and women, clinicians can avoid delays in diagnosis, tailor therapeutic approaches more effectively, and ultimately improve long-term prognosis and quality of life for patients with PsA.

To better address sex-based differences in psoriatic arthritis, prospective, sex-stratified clinical trials are necessary to assess how therapies perform across sexes. These trials should systematically assess treatment responses and investigate how sex and gender influence disease progression and outcomes. In parallel, clinical practice could benefit from incorporating actionable, sex-informed strategies. For example, routine hormonal profiling—including serum levels of estrogen, progesterone, and testosterone—could be incorporated at the baseline and at key disease milestones (e.g., disease flare, treatment resistance, menopausal transition) to identify hormonal influences on disease activity and guide therapeutic selection, particularly in women with fluctuating hormonal states or men with hypogonadism. Additionally, pain sensitivity assessments using validated instruments [93] could be routinely administered alongside disease activity scores. These tools can help differentiate between inflammatory and non-inflammatory pain and identify sex-based differences in pain perception that may otherwise be overlooked in conventional disease activity measures. Further research in this area would not only deepen our understanding of sex-based disparities in PsA but also pave the way for more personalized, equitable treatment approaches that improve outcomes for all patients.

## Figures and Tables

**Table 1:** Summary of the main sex differences in PsA concerning clinical presentation, treatment response, and comorbidities. Abbreviations: BMI—body mass index; DIP—distal interphalangeal.

	Males	Females
Clinical presentation and radiological involvement	More frequent axial involvement [22,23] Oligoarticular pattern/DIP involvement [22,23] More severe radiographic damage [22,23] More extensive cutaneous disease [22,23]	More frequent peripheral arthritis [22,23] Polyarticular pattern [22,23] Increased pain intensity [22,23] Greater functional impairment [22,23]
Cardiovascular risk (dyslipidemia, hypertension)	Mixed data on prevalence [24] Tendency toward higher prevalence in men [25,26]	Mixed data on prevalence [24]
Obesity and metabolic syndrome	Mixed data on prevalence [24]	Mixed data on prevalence [24] Lower treatment response rates with higher BMI [27]
Psychiatric burden	Lower rates of psychiatric comorbidities compared to women [22]	Depression and fibromyalgia are more frequently observed, influencing pain perception and overall quality of life [22]
Treatment response	Better response to biological therapies with higher remission rates and longer treatment persistence [28,29,30]	Poorer response to biological therapies (particularly TNF inhibitors) with lower remission rates and shorter treatment persistence [28,29,30]

**Table 2:** Summary of the main studies evaluating various therapeutic agents in PsA and the associated sex-related differences in primary outcomes. RWD—real-world data; CI—confidence interval; aHR—adjusted hazard ratio; RR—relative risk; ACR20/50/70 20%, 50%, or 70% improvement from baseline in the American College of Rheumatology criteria; cDAPSA—clinical Disease Activity Index for Psoriatic Arthritis; HAQ-DI—Health Assessment Questionnaire Disability Index, LDA—low disease activity; MDA—minimal disease activity; PsAID-12—total PsA Impact of Disease-12; RCTs—randomized controlled trials; TNFi—TNF-alpha inhibitors; IL-17i—IL-17 inhibitors; IL-12/23i—IL-12/23 inhibitors; JAKi—Janus kinase inhibitors.

Therapeutic Agent	Research	Primary Outcome	Women	Men
TNFi and Il-12/23i (ustekinumab) TNFi	Van Kuijk 2023 [33]—RWD Pina Vegas 2023 [28]— Cohort study Hellamand 2024 [29]— RWD	Comparing cDASPSA, HAQ-DI, PsAID-12 at 6 and 12 mo. 95% CI Treatment persistence rates at 1, 2, and 3 years aHR = 1.4 (99%CI, 1.3–1.5) LDA at 6 months RR = 0.82 (95% CI, 0.80–0.84)	At 6 months cDAPSA = 32.3 HAQ-DI = 1.3 PsAID-12 = 6.0 At 12 months LDA = 57.8% MDA = 33.7% HAQ-DI = 0.85 PsAID-12 = 3.5 49.3%, 33.6%, 25.7% 64%	At 6 months cDAPSA = 26.8 HAQ-DI = 0.93 PsAID-12 = 5.1 At 12 months LDA = 80.3% MDA = 55.5% HAQ-DI = 0.50 PsAID-12 = 2.4 60.9%, 47.1%, 39.8% 78%
IL-17i	Eder 2022 [27]—post hoc analysis of 2 RCTs Pina Vegas 2023 [28]	ACR20/50/70; MDA/VLDA; DAPSA LDA/remission at week 156 Treatment persistence rates at 1, 2, and 3 years aHR = 1.2 (99%CI, 1.1–1.3)	56.0%/38.3%/24.2% 26.8%/10.2% 48.4%/16.8% 56.2%, 38.5%, 28.4%	68.8%/53.8%/32.3% 44.9/%21.5% 62.7%28.4% 64.7%, 47.5%, 38.7%
IL 12/23i	Pina Vegas 2023 [28]	Treatment persistence rates at 1, 2, and 3 years aHR = 1.1 (99%CI, 0.9–1.3)	60.7%, 40.5%, 31.0%	66.5%, 48.0%, 40.0%
JAKi	Pina Vegas 2023 [28] Eder 2023 [30]—post hoc analysis of 3 RCTs	Treatment persistence rates at 1, 2, and 3 years aHR = 1.2 (99%CI, 0.9–1.6) ACR20/50/70 response	38.9%, 22.3%, 12.3% Comparable results	45.9%, 21.9%, 11.6% Comparable results

## Supplementary Materials

The following supporting information can be downloaded at https://www.mdpi.com/article/10.3390/jcm14124124/s1, Table S1. Key characteristics of each included study. Table S2. Quality assessment of all studies evaluated using an abbreviated version of the Standard Quality Assessment Criteria for Evaluating Primary Research Papers from a Variety of Fields developed by Kmet et al.

## Abbreviations

The following abbreviations are used in this manuscript:

BMI	body mass index
bDMARDs	biologic disease-modifying antirheumatic drugs
CASPAR	classification Criteria for Psoriatic Arthritis
CRP	C-reactive protein
DAS-28	disease activity score using 28 joint counts
cDAPSA	clinical Disease Activity Index for Psoriatic Arthritis
csDMARDs	conventional synthetic disease-modifying antirheumatic drugs
DIP	distal interphalangeal
HAQ-DI	Health Assessment Questionnaire Disability Index
HLA-B27	human leukocyte antigen-B27
(IFN-γ)	interferon-gamma
IL-17i	IL-17 inhibitors
IL-12/23i	IL12/23 inhibitors
IL-23p19i	IL-23p19 inhibitors
IL-6	interleukin-6
IL-17	interleukin-17
IL-23	interleukin-23
JAKi	Janus kinase inhibitors
KIR	killer cell immunoglobulin-like receptors
LDA	low disease activity
LEI	Leeds Enthesitis Index
MASES	Maastricht Ankylosing Spondylitis Enthesitis Score
MACEs	major adverse cardiovascular events
MDA	minimal disease activity
MeSH	Medical Subject Headings
MRI	magnetic resonance imaging
mSS	modified Steinbrocker score
mSASSS	modified Stokes Ankylosing Spondylitis Spine Score
NSAIDs	nonsteroidal anti-inflammatory drugs
PDE4i	phosphodiesterase 4 inhibitors
PROs	patient-reported outcomes
PsC	psoriasis without PsA
PtGA	Patient Global Assessment
PRISMA	Preferred Reporting Items for Systematic Reviews and Meta-Analyses
PASI	Psoriasis Area and Severity Index
PsA	psoriatic arthritis
PsAID-12	Psoriatic Arthritis Impact of Disease-12
RA	rheumatoid arthritis
SpA	spondyloarthritis
RWD	real-world data
Th1	T helper 1
TJC	tender joint count
TNF-α	tumor necrosis factor-alpha
TNFi	tumor necrosis inhibitors
Treg	regulatory T lymphocytes
tsDMARDS	targeted synthetic disease-modifying antirheumatic drugs
VAS	visual analog scale
