# Supplementary material for: Unveiling Sex-Related Variability in Psoriatic Arthritis: A Call for Personalized Care
Source: J Clin Med. 2025 Jun 11;14(12):4124. doi: 10.3390/jcm14124124 (PMC12193862; doi:10.3390/jcm14124124)
Supplement: Supplementary file 1 [file jcm-14-04124-s001.zip › jcm-3639686-supplementary.pdf]

Supplementary Materials

Search Strategies

Note: The search strategies were based primarily on keyword searches using Boolean operators. While MeSH terms were considered, they were not systematically applied. The following reflects the practical search approach conducted across the three databases.

PubMed Search Strategy (as conducted)

(psoriatic arthritis) AND (sex differences OR gender OR male OR female)

AND (radiographic OR treatment OR comorbidities)

Filters applied: English language, adult population, publication date from 2000 to 2025

Embase Search Strategy (as conducted)

('psoriatic arthritis') AND ('sex differences' OR 'gender' OR 'male' OR 'female')

AND ('radiographic' OR 'treatment' OR 'comorbidities')

Limits: English, Adults (≥18 years), 2000–2025

Cochrane Library Search Strategy (as conducted)

(psoriatic arthritis):ti,ab,kw

AND (sex differences OR gender OR male OR female):ti,ab,kw

AND (radiographic OR treatment OR comorbidities):ti,ab,kw

Publication Years: 2000 to 2025

These search strings reflect the actual process undertaken in this review and are provided for transparency and reproducibility.

Table S1. Key characteristics of each included study.

	Country	Design	Sample size	Outcomes/main findings
Koc 2025	Netherlands	Observational longitudinal cohort study	415	Female RA patients have higher disease activity, need more bDMARDs and experience greater functional decline, highlighting the need for sex-specific treatment.
Coates 2023	Multicentric	Systematic review	27 studies; population size varied across the included studies, from 108 patients to 8677 patients	Women had poorer responses to treatment, indicated by outcome measures such as ACR responses and minimal disease activity.
Eder 2013	Multicentric (America, Asia, Europe)	Cross-sectional observational study	590	Men with PsA are more likely to develop axial involvement and radiographic joint damage, while women are more likely to report limitation in function and impaired quality of life.
Atzeni 2024	Multicentric	Narrative review	NA	Men with PsA: more dyslipidemia, cardiovascular disease, and gout;<br>Women with PsA: more lung disease, depression, hypothyroidism, osteoporosis, and osteoarthritis;<br>Cardiovascular risk: elevated in PsA overall; sex differences are inconsistent across studies.
Eder 2022	Multicentric (America, Asia, Europe)	Post-hoc analysis of 2 RCTs	SPIRIT-P1 417<br>SPIRIT-P2 363	Greater treatment response rates in males

Pina Vegas 2023	France	Cohort study	14778	Better treatment persistence rates at 1, 2 and 3 years in males for TNFi and IL-17i.
Hellamand 2024	Europe	RWD	7679	Greater treatment response rates in men.
Eder 2023	Multicentric	Posthoc analysis of 3 RCTs	816	Comparable treatment responses between men and women.
Eder 2012	Multicentric	Narrative review	NA	Men: more axial disease and greater radiographic damage Women: more peripheral joint involvement, higher tender joint counts, worse quality of life, and earlier TNFi discontinuation.
Van Kuijk 2023	Europe	RWD	929	Lower treatment response and persistence rates in women compared to men.
Braaten 2019	America (Utah)	RWD	253	Women with PsA experience greater fatigue, pain and functional limitation.
Mease 2017	USA	RWD	1567	Patients with versus without dactylitis or enthesitis had greater disease activity.
Duruoz 2021	Turkey	Multicentric, cross-sectional observational study	1003	Female patients tend to have a more severe disease course.
Queiro 2001	Spain	Retrospective cross-sectional study	100	Men are more likely to have isolated axial disease associated with HLA-B27 positivity Women exhibit more severe peripheral joint involvement
Tarannum 2022	Multicentric (Canada, Singapore, USA)	Narrative review	NA	Women with PsA have higher disease burden and worse treatment outcomes than men.
Gupta 2021	Multicentric	Systematic review and meta-analysis	39 studies 158797 patients	Comorbidities, especially cardio-metabolic, are highly

				prevalent in PsA and more common than in healthy controls.
Hojgaard 2016	Denmark, Iceland	RWD	1388	Obesity reduces TNFi response and adherence in PsA, especially in men.
Menis 2023	France	Retrospective observational single-center study	132	Women with PsA had more enthesitis, obesity, and DMARD use; men had more axial disease and nail changes.
Landgren 2024	Sweden	Post-hoc analysis of a prospective interventional study	41	Women with PsA had higher IL-23 and leptin levels, which improved after weight loss; changes were less pronounced in men.
Albrecht 2023	Germany	Retrospective observational study	11,984 individuals with PsA 119,840 age- and sex-matched individuals	Polypharmacy and comorbidities were more common in PsA, especially among older adults and women
McDonough 2014	Canada	Cross-sectional observational study	306 PsA patients 135 PsC patients	Depression and anxiety were more common in PsA than psoriasis alone, especially in women and those with greater disease activity
Kostis 2012	Greece	Cross-sectional observational study	83 PsA patients<br><br>199 RA patients	Anxiety and illness concerns lower physical quality of life in PsA, highlighting the need to address mental health in care
Mease 2018	USA	Retrospective observational study	148 MDA Achievers: 34 MDA Non-Achievers: 114	Patients achieving minimal disease activity had lower tender joint counts, less pain and fatigue, and better physical function than non-responders.

Mease 2017	International, multicentric study	RCT	417	In biologic-naive patients with active PsA, ixekizumab significantly improved disease activity, physical function, and inhibited structural damage progression, with a safety profile consistent with previous studies.
Nash 2017	Multicentric	RCT	363	Ixekizumab significantly improved signs and symptoms of PsA in patients who had an inadequate response to TNFi therapy, with a safety profile consistent with previous studies.
Lesuis 2012	Sweden	RWD	4,493	Women with RA or psoriasis reported worse subjective disease activity measures than men, despite similar objective disease activity.

NA – Not Applicable, RWD - real world data, RCT- Randomized controlled trial, RA- rheumatoid arthritis, bDMARDs- biologic disease-modifying antirheumatic drugs, ACR- American College of Rheumatology, PsA- Psoriatic Arthritis; PsC- psoriasis (cutaneous), TNFi- Tumor necrosis factor inhibitors, MDA- minimal disease activity

Table S2. Quality assessment of all studies evaluated using an abbreviated version of the Standard Quality Assessment Criteria for Evaluating Primary Research Papers from a Variety of Fields developed by Kmet et al.

	Q1	Q2	Q3	Q4	Q5	Q6	Q7	Q8	Q9	Q10	Q11	Q12
Koc 2025	Y	Y	Y	Y	NA	NA	NA	Y	Y	Y	Y	Y
Coates 2023	Y	Y	Y	NA	NA	NA	NA	Y	NA	Y	Y	Y
Eder 2013	Y	Y	Y	Y	NA	NA	NA	P	Y	Y	Y	Y
Atzeni 2024	Y	Y	P	P	NA	NA	NA	Y	Y	Y	Y	Y
Eder 2022	Y	Y	Y	Y	NA	NA	NA	Y	Y	Y	Y	Y
Pina Vegas 2023	Y	Y	Y	Y	NA	NA	NA	Y	Y	Y	Y	Y
Hellamand 2024	Y	Y	Y	Y	NA	NA	NA	Y	Y	Y	Y	Y
Eder 2023	Y	Y	Y	Y	Y	Y	Y	P	Y	Y	Y	Y
Eder 2012	Y	Y	N	P	NA	NA	NA	P	NA	NA	P	P
Van Kuijk 2023	Y	Y	Y	Y	NA	NA	NA	P	Y	Y	Y	Y
Braaten 2019	Y	Y	Y	P	NA	NA	NA	Y	P	P	Y	Y
Mease 2017	Y	Y	Y	Y	NA	NA	NA	Y	Y	Y	Y	Y
Duruoz 2021	Y	Y	Y	Y	NA	NA	NA	Y	Y	Y	Y	Y
Queiro 2001	Y	Y	P	Y	NA	NA	NA	P	Y	Y	Y	Y
Tarannum 2022	Y	Y	NA	NA	NA	NA	NA	Y	NA	NA	Y	Y
Gupta 2021	Y	Y	Y	Y	NA	NA	NA	Y	Y	Y	Y	Y
Hojgaard 2016	Y	Y	P	Y	NA	NA	NA	Y	Y	Y	Y	Y
Menis 2023	Y	Y	P	P	NA	NA	NA	Y	Y	Y	Y	Y
Landgren 2024	Y	Y	Y	Y	NA	NA	NA	Y	P	Y	Y	Y
Albrecht 2023	Y	Y	Y	Y	NA	NA	NA	Y	Y	Y	Y	Y
McDonough 2014	Y	Y	Y	Y	NA	NA	NA	Y	Y	Y	Y	Y
Kostis 2012	Y	Y	P	P	NA	NA	NA	Y	Y	Y	Y	Y
Mease 2018	Y	Y	Y	Y	NA	NA	NA	Y	Y	Y	Y	Y
Mease 2017	Y	Y	Y	Y	Y	Y	Y	Y	Y	Y	Y	Y
Nash 2017	Y	Y	Y	Y	Y	Y	Y	Y	Y	Y	Y	Y
Lesuis2012	Y	Y	Y	P	NA	NA	NA	Y	Y	Y	Y	Y

Q1 Question or objective sufficiently described?, Q2 Design appropriate to answer study questions?, Q3 Were the source of information appropriate and well described? Q4 Were the units of participation sufficiently described? Q5 If random allocation to treatment group was done, is it well described? Q6 If interventional and blinding of investigators to intervention present, is it well described? Q7 If interventional and blinding of subjects to intervention present, is it well described? Q8 Were the means of assessment reported? Q9 Sample size was appropriate? Q10 Were analytic methods well described and appropriate? Q11 Controlled for confounding? Q12 Are the conclusions supported by the results? Y – Yes, P - Partial, N – No, NA – Not Applicable
